# Structural Equation Modeling (SEM) of Cysticercosis in School-Aged Children in Tibetan Rural Farming Areas of Western China: Implications for Intervention Planning

**DOI:** 10.3390/ijerph16050780

**Published:** 2019-03-04

**Authors:** Huan Zhou, Qingzhi Wang, Junmin Zhou, Tiaoying Li, Alexis Medina, Stephen A. Felt, Scott Rozelle, John J. Openshaw

**Affiliations:** 1 West China School of Public Health, Sichuan University, Chengdu 610041, China; zhouhuan@scu.edu.cn (H.Z.); 13183812895@163.com (Q.W.); 2 Institute of Parasitic Diseases, Sichuan Centers for Disease Control and Prevention, Chengdu 610041, China; litiaoying@sina.com; 3 Freeman Spogli Institute for International Studies, Stanford University, Stanford, CA 94305, USA; amedina5@stanford.edu (A.M.); rozelle@stanford.edu (S.R.); 4 Department of Comparative Medicine, Stanford University School of Medicine, Stanford, CA 94305, USA; felt@stanford.edu; 5 Division of Infectious Diseases and Geographic Medicine, Department of Medicine, Stanford University, Stanford, CA 94305, USA; jjo@stanford.edu

**Keywords:** neurocysticercosis, school-aged children, intervention planning, structural equation model

## Abstract

Neurocysticercosis (NCC) significantly contributes to morbidity in developing countries. We recently published a study of prevalence and risk factors in school-aged children in three mountainous areas in Sichuan province of western China. Using structural equation modeling (SEM) on data from that study to guide intervention planning, here we examine risk factors grouped into three broad interventional categories: sociodemographics, human behavior, and sources of pork and pig husbandry. Because neuroimaging is not easily available, using SEM allows for the use of multiple observed variables (serological tests and symptoms) to represent probable NCC cases. Data collected from 2608 students was included in this analysis. Within this group, seroprevalence of cysticercosis IgG antibodies was 5.4%. SEM results showed that sociodemographic factors (*β* = 0.33, *p* < 0.05), sources of pork and pig husbandry (*β* = 0.26, *p* < 0.001), and behavioral factors (*β* = 0.33, *p* < 0.05) were all directly related to probable NCC in school-aged children. Sociodemographic factors affected probable NCC indirectly via sources of pork and pig husbandry factors (*β* = 0.07, *p* < 0.001) and behavioral variables (*β* = 0.07, *p* < 0.001). Both sociodemographic factors (*β* = 0.07, *p* < 0.05) and sources of pork and pig husbandry factors (*β* = 0.10, *p* < 0.01) affected probable NCC indirectly via behavioral variables. Because behavioral variables not only had a large direct effect but also served as a critical bridge to strengthen the effect of sociodemographics and sources of pork and pig husbandry on probable NCC, our findings suggest that interventions targeting behavioral factors may be the most effective in reducing disease.

## 1. Introduction

Caused by the zoonotic tapeworm *Taenia solium*, cysticercosis affects millions of people living in Asia, Africa, and Latin America [1]. There are two types of human infection: intestinal taeniasis and cysticercosis. Intestinal taenisasis is caused when humans consume uncooked or under-cooked pork containing cysticerci. The adult tapeworm develops and inhabits the gastrointestinal tract, with humans serving as the definitive host [2]. Cysticercosis is acquired by ingestion of oncospheres released in the feces of humans with intestinal taeniasis [3]. Hexacanths migrate to tissue throughout the body, forming cysticerci. In the case of cysticercosis, humans are a dead-end intermediate host for the tapeworm [4,5,6]. Human cysticercosis has been linked to poor sanitation, free-range pig raising, and limited pork inspection and control [7,8].

Neurocysticercosis (NCC), the most severe form of cysticercosis, occurs when *T. solium* metacestodes invade the brain causing a wide range of neurologic morbidity [4,9,10] including seizures, chronic headaches, psychiatric disturbances, cognitive impairment, and focal neurologic deficits [2,11,12,13]. While the worldwide burden of NCC is not fully appreciated, it is a leading cause of death from food-borne disease resulting in 2.8 million disability-adjusted life years lost in 2010 [8].

In China, NCC is prevalent in almost every province and autonomous region, with a high density in Southwestern China [14,15,16]. It is estimated that approximately 7 million people in China are affected by *T. solium* neurocysticercosis [1]; however, the risk factors and impact of NCC in rural areas of Southwest China remain poorly characterized [3,17]. A better understanding of the epidemiology and risk factors of the disease in these rural areas is needed to inform intervention strategies.

The prevalence and effects of NCC among school-aged children have received little scientific attention. We found the seroprevalence of human cysticercosis IgG antibodies to be 6% in 5th and 6th graders overall and higher than 20% in some schools [16]. Owning pigs, feeding pigs human feces, and reporting proglottids in feces were found to be risk factors for the presence of antibodies [16].

In the present manuscript, we use structural equation modeling (SEM) [18,19,20] to categorize important areas for intervention in pediatric populations. We use SEM to group factors into three latent variable groups, each representing potential intervention categories. Because neuroimaging is neither easily available in the study area nor financially feasible for large field surveys, using SEM allows for the use of multiple observed variables (serological tests and symptoms) to represent an unobserved construct: probable NCC cases.

## 2. Materials and Methods

Detailed field and laboratory methodologies for this study have been described elsewhere [16], and are only briefly reviewed here.

### 2.1. Study Site and Sampling

This study was conducted in three rural prefectures (Aba, Ganzi, Liangshan) in Sichuan province. Located at the eastern extremity of the Tibetan Plateau and with an average altitude of 2700 meters, these areas are largely inhabited by poor smallholder Tibetan farmers, many of whom raise pigs. We utilized a school-based sampling technique, focusing on fifth and sixth grade students [16].

### 2.2. Data Collection

#### 2.2.1. Ethics

The study received ethical approval from the Stanford University institutional review board (Protocol ID 35415) and from the Sichuan University Medical Ethical Review Board (K2015031). The school principals, who are the children’s legal guardians while in school, provided consent for student participation and each student provided verbal assent prior to participating.

#### 2.2.2. Questionnaire Survey

Questionnaires including a student questionnaire and a take-home head of household questionnaire were used to collect information on sociodemographic factors, behavioral factors, sources of pork and pig husbandry, and neurologic symptoms [16].

#### 2.2.3. Serological Examination

At each school, approximately 5 mL of blood was collected from each participating student via venous puncture. Sera were tested for *T. solium* human cysticerosis IgG antibodies using an enzyme-linked immunosorbent assay (ELISA) based on low-molecular-weight antigens (LMWAgs) of *T. solium* cysticerci collected from pigs in Chinese endemic areas. LMWAg-based assays have been shown to be highly sensitive and specific [21,22], have been used in previous field studies [23], and are especially attractive given their low cost, quantifiable results, and simplicity for use in low-resource areas [22]. Detailed assay methodology has been published previously [16,22].

### 2.3. Statistical Analysis

Because the timing of the survey conflicted with the class time of students, some schools in Ruoergai county did not complete the questionnaire, and 268 students in Ruoergai county were removed from the analysis because the questionnaire was not completed. In cases of missing data, we applied a conditional mode to fill in missing values [24].

Univariate analysis was conducted to depict the distribution of all factors. The associations between sociodemographics, behavioral variables, sources of pork and pig husbandry, and probable NCC were examined by SEM. Two steps were taken when building our model. First, to assess whether observed variables (e.g., age, sex, and reported behaviors) were adequate indicators of latent variables (not directly observed but estimated from directly measured variables) we conducted confirmatory factor analysis to test associations. Second, the structural model was carried out to estimate the strengths of the associations among latent variables.

**Figure 1:**
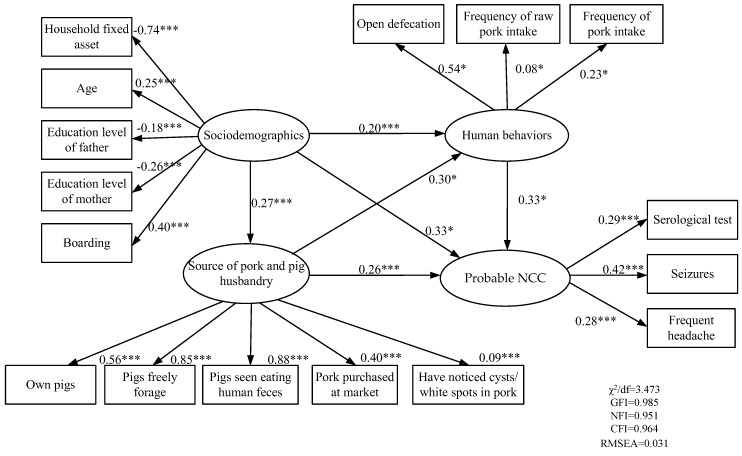
Structural equation model of probable NCC in school-aged children in Tibetan rural farming areas of western China. Note: Values indicate standardized coefficients; * *p* < 0.05, *** *p* < 0.001.

In our SEM (Figure 1), we propose that risk of NCC is associated with three latent variables: sociodemographics, human behavior, and sources of pork and pig husbandry. The sociodemographics latent variable was estimated by age, sex, grade, boarding status (living and sleeping at school versus returning home to sleep), parental education level, and household asset score. A household asset score was developed using principal component analysis to aggregate all asset ownership variables. The behavioral latent variable was estimated by the reported presence of open defecation, overall frequency of pork consumption, and frequency of raw pork consumption. The sources of pork and pig husbandry latent variable was estimated by reports of purchasing pork at markets, pig ownership, herd husbandry practices (allowing free foraging, feeding pigs human feces, and if disposal methods left feces accessible to pigs), and if households reported noticing cysts consistent with cysticerci in their pork in the previous 5 years (photos of cysticerci in pork were presented to participants). For the latent variable representing probable NCC, we selected factors based on China’s national diagnostic standard [25] including the presence of IgG antibodies to *T. solium* cysticercosis, the presence of seizures, and the presence of frequent headaches (defined here as self-reported occurring 6 times a month or more).

Multiple indicators were used to evaluate the fit of the model, including χ^2^/degrees of freedom (χ^2^/df), goodness-of-fit index (GFI), normed fit index (NFI), comparative fit index (CFI), parsimony goodness-of-fit index (PGFI), parsimony normed fit index (PNFI), parsimony comparative fit index (PCFI), root mean square residual (RMR), and root mean square error of approximation (RMSEA).

The association was considered to be statistically significant if the 2-sided *p* value was less than 0.05. All analyses were performed using SPSS (IBM, Armonk, NY, USA) and AMOS 21.0 statistical software (IBM, Armonk, NY, USA) [26].

## 3. Results

A total of 2608 students from the data analyzed in our original prevalence manuscript [16] were included in the SEM analysis.

### 3.1. Summary of Observed Variables

Table 1 summarizes descriptive statistics for the variables utilized in the SEM.

#### 3.1.1. Sociodemographics

Student ages ranged from 8 to 20 years old (mean age of 12.1), and 48.4% of students were male. The majority boarded at their schools, with 61.7% of students reporting sleeping at school. Of the parents, 59.5% of mothers and 36.0% of fathers never attended school.

#### 3.1.2. Behavioral Variables

Just under half of participants (47.5%) reported open defecation. Almost all (97.1%) reported consuming pork once or more in the month preceding the questionnaire administration, although 84.2% reported never consuming raw pork. 

#### 3.1.3. Sources of Pork and Pig Husbandry Variables

Among students, 43.0% reported that their pigs freely foraged, and 43.8% indicated that their pigs consumed human feces. Only 27.5% of participants reported consuming market-purchased pork. Twenty-eight percent reported noting white spots or cysts in pork at some time in the five years preceding the survey.

#### 3.1.4. Probable Neurocysticercosis (NCC)

Overall, 5.4% of all participants had positive serological test result; 3.6% had seizures; while 8.7% complained of frequent headaches (6 or more episodes per month). 

### 3.2. Structural Equation Model

#### 3.2.1. Associations between Latent Variables and Indicator Variables

The model (Figure 1) fit well with the data (RMSEA = 0.031, NFI = 0.951, GFI = 0.985, CFI = 0.964, and χ^2^/df = 3.473). Probable NCC could be well−represented by the serological test results (*β* = 0.29, *p* < 0.001), having seizures (*β* = 0.42, *p* < 0.001), and frequent headaches (*β* = 0.28, *p* < 0.001). Age (*β* = 0.25, *p* < 0.001), boarding (*β* = 0.40, *p* < 0.001), educational level of father (*β* = −0.18, *p* < 0.001), educational level of mother (*β* = −0.26, *p* < 0.001), and household fixed asset score (*β* = −0.74, *p* < 0.001) were good indicators of sociodemographics. For behavioral variables, open defecation (*β* = 0.54, *p* < 0.05), frequency of pork consumption (*β* = 0.23, *p* < 0.05), and frequency of raw pork consumption (*β* = 0.08, *p* < 0.05) were adequate indicators. Pork purchased at market (*β* = 0.40, *p* < 0.001), pig ownership (*β* = 0.56, *p* < 0.001), households allowing pigs to freely foraging (*β* = 0.85, *p* < 0.001), pigs consuming human feces (*β* = 0.88, *p* < 0.001), and noting white spots or cysts in pork (*β* = 0.09, *p* < 0.001) were used to represent sources of pork and pig husbandry variables.

#### 3.2.2. Associations between Latent Variables

Sociodemographic variables (*β* = 0.33, *p* < 0.05), sources of pork and pig husbandry (*β* = 0.26, *p* < 0.001), and behavioral variables (*β* = 0.33, *p* < 0.05) were all direct risk factors for probable NCC among children.

Sociodemographic factors could affect the likelihood of being categorized as probable NCC indirectly via sources of pork and pig husbandry (*β* = 0.27, *p* < 0.001) and behavioral variables (*β* = 0.20, *p* < 0.001). Sources of pork and pig husbandry (*β* = 0.26, *p* < 0.001) could affect the likelihood of being categorized as probable NCC indirectly via behavioral variables. The effect size depicted by squared multiple correlations (R^2^) was 0.46.

### 3.3. Effects of Sociodemographics, Behavioral Variables and Sources of Pork and Pig Husbandry on Probable NCC

Table 2 indicates the total effect of all latent variables on probable NCC. The total effect of a given variable group is the sum of its direct and indirect effects. Sociodemographics and behavior variables have the largest direct effect on probable NCC with the same standardized regression coefficient of 0.33. Indirect effect refers to the effect of one variable on another via a mediator (e.g., sociodemographics → behavioral variables → probable NCC), and is the product of the two individual direct effects. For example, the indirect effect of sociodemographics on probable NCC via behavioral variables was 0.07 (0.20 × 0.33), via sources of pork and pig husbandry was 0.07 (0.27 × 0.26), and via both sources of pork and pig husbandry and behavioral variables was 0.03 (0.27 × 0.30 × 0.33). The total indirect effect on probable NCC from sociodemographic variables is obtained by summing all indirect effects (0.07 + 0.07 + 0.03 = 0.13). Although sociodemographics had the largest total effect after adding the indirect effect, the behavior variables played a more key role in the model for large direct effect and strengthening all other variables with probable NCC.

## 4. Discussion

This study systematically examines the risk factors associated with NCC using SEM. Our analysis used a latent variable, consisting of serologic test results and children’s self-reported symptoms, to classify students as probable neurocysticercosis cases. All three of the latent variables representing general risk factor categories, which included sociodemographics, sources of pork and pig husbandry, and behavioral variables, contributed to children being characterized as probable NCC.

Our results indicate that sociodemographics and behavior had the same direct effect on probable NCC. After adding the indirect effects via behavior, sociodemographics represented the largest total effect in our model. This was followed by the latent variable representing sources of pork and pig husbandry, which affected classification directly as well as indirectly via behavior. However, behavioral variables not only had a large direct effect, but also served as a bridge to strengthen the effect of sociodemographics and sources of pork and pig husbandry on probable NCC.

Our sociodemographic latent variable included household income, parental education, age of the student, and school boarding status. Sociodemographic factors have been identified as risk factors for cysticercosis in the existing literature. Previous studies have identified lower levels of education as a significant risk factor for human cysticercosis [27]. Prior research in China also supports the importance of sociodemographic factors, with a study conducted in Gansu province showing that older age was associated with higher risk of cysticercosis among children [28] and other research demonstrating that poor households in China were more likely to report cysticercosis [28,29,30]. Our own work links the sociodemographic variables of parental education and boarding to the likelihood of a child receiving treatment for gastrointestinal worms, with children who do not board at school and have more educated parents being more likely to receive treatment for gastrointestinal worms [16]. The importance of sociodemographics in our SEM model suggests that interventions should be prioritized. Specifically, children in poor households, parents with low education levels, and students boarding at schools should receive more attention.

The latent variable representing sources of pork and pig husbandry was significantly associated with probable NCC, suggesting that this would be an impactful area for intervention. Pig ownership, the consumption of home-raised pigs, the presence of freely foraging pigs, and pigs having access to human feces were identified as significant risk factors in our study. The link between pig ownership and human cysticercosis has also been widely reported in studies in Africa and South America [31,32]. Similar to our findings, other studies have demonstrated that consuming market-purchased pork decreases the risk of cysticercosis [31], suggesting that pork sold at markets is more likely to come from pigs which lack the risk factors for *T. solium* infection. Pigs being allowed to freely roam and having easy access to human feces have been widely recognized as a risk factor for pigs becoming infected [32,33,34,35]. Our SEM suggests interventions related to pig husbandry and meat sourcing such as porcine vaccination, the treatment of pigs with anthelminthic medications as well as improved meat inspection [36] would likely be impactful in our area of study.

Behavioral variables not only had a large direct effect, but also served as a critical bridge to strengthen the effect of sociodemographics and sources of pork and pig husbandry on probable NCC. These findings suggest that interventions targeting behaviors may be the most effective in decreasing disease. Similar to our work, open defecation and consuming raw pork are widely recognized as risk factors for disease [3,7,8,29]. Our SEM suggests interventions related to such behaviors would have a large impact. Furthermore, compared with the sociodemographics and sources of pork and pig husbandry, behavioral variables may be easier to change in our population, and may be more easily targeted by school-based educational campaigns than the other areas of intervention. Therefore, health interventions could educate children to regularly use toilets and avoid consuming raw pork.

This study has several limitations. The use of cross-sectional data does not allow us to infer causality. The use of self-report data can potentially introduce recall bias especially for variables based on participants’ long-term memory such as having seizures during the last year. In addition, since this study was conducted in extremely remote and poor mountainous areas with limited medical resources, we were not able to obtain neuroimaging allowing for a definite diagnosis of NCC, instead having to rely on the SEM to model this outcome.

## 5. Conclusions

The strength of using SEM to better understand interventions directed at *T. solium* cysticercosis is the ability to create latent variables representing broad classes of intervention as well as the unobserved construct of probable NCC cases. Our results suggest that future intervention programs aimed at pediatric populations in our study area would be most effective if they are considering sociodemographic factors, sources of pork and pig husbandary, and especially behavioral factors. Our results suggest that an intervention package consisting of stopping open defecation and consuming raw pork, as well as treatment of pigs utilizing vaccination and anthelminthic medications, particularly in pediatric populations boarding at schools and in poor households may be the most impactful in our study area.

## Figures and Tables

**Table 1:** Descriptive statistics for the variables utilized in the structural equation modeling (SEM) (*n* = 2608).

Latent Variable	Indicator Variable		Number	Percentage (%)
Sociodemographic variables	Age	11 and under	927	35.5
		12	833	31.9
		13	507	19.4
		14 and above	341	13.1
	Sex	Male	1264	48.4
	Grade	5	1394	53.5
	Boarding at school		1610	61.7
	Highest education level attained: father	Unknown	353	13.5
		Never attended school	940	36.0
		Primary school	987	37.8
		Middle school and above	328	12.6
	Highest education level attained: mother	Unknown	264	10.1
		Never attended school	1533	59.5
		Primary school	648	24.8
		Middle school and above	143	5.5
	Household asset score	1st Quartile (Poorest)	725	27.8
		2nd Quartile	698	26.8
		3rd Quartile	601	23.0
		4th Quartile (Wealthiest)	584	22.4
Behavioral variables	Reports open defecation		1238	47.5
	Frequency of pork consumption	Never	76	2.9
		Rarely (1–2 times/month)	517	19.8
		Sometimes (3–5 times/month)	520	19.9
		Often (6–10 times/moths)	692	26.5
		Always (more than 10 times/month)	803	30.8
	Frequency of raw pork consumption	Never	2195	84.2
		Rarely (1–2 times/month)	297	11.4
		Sometimes (3–5 times/month)	80	3.1
		Often (6–10 times/moths)	25	1.0
		Always (more than 10 times/month)	11	0.4
Sources of pork and pig husbandry	Pig ownership		1834	70.3
	Pigs allowed to freely forage		1121	43.0
	Pigs consume human feces		1143	43.8
	Pork purchased at market		717	27.5
	Have noticed white spots in pork in the previous 5 years		741	28.4
Probable neurocysticercosis (NCC)	T. solium cysticercosis IgG positive		140	5.4
	Having seizures		93	3.6
	Frequent headaches (≥6/month)		226	8.7

**Table 2:** Effects of sociodemographics, behavioral variables and sources of pork and pig husbandry on probable NCC.

Latent Variable	Direct Effect	Indirect Effect	Total Effect
Sociodemographics	0.33	0.13	0.46
Sources of pork and pig husbandry	0.26	0.10	0.36
Behavioral variables	0.33	0.00	0.33
